# Alexithymia, Self-Compassion, Emotional Resilience, and Cognitive Emotion Regulation: Charting the Emotional Journey of Cancer Patients

**DOI:** 10.3390/curroncol30100641

**Published:** 2023-09-28

**Authors:** Ipek Ozonder Unal, Cetin Ordu

**Affiliations:** 1 Department of Psychiatry, Tuzla State Hospital, Içmeler Mahallesi, Istanbul 34947, Turkey; 2 Department of Medical Oncology, Gayrettepe Florence Nightingale Hospital, Istanbul 34381, Turkey; cetin.ordu@demiroglu.bilim.edu.tr

**Keywords:** anxiety, depression, emotion regulation, psychological resilience, psycho-oncology, self-compassion

## Abstract

Cancer’s profound impact on emotional well-being necessitates an exploration into the underlying psychological mechanisms influencing depression and anxiety in patients. In this study, we explored the potential role of self-compassion, alexithymia, and cognitive emotion regulation mechanisms in influencing depressive and anxiety symptoms among cancer patients. A total of 151 stage 4 cancer patients participated. Instruments applied included the Beck Depression Scale (BDS), Beck Anxiety Inventory (BAI), Self-Compassion Scale (SCS), Cognitive Emotion Regulation Scale (CERQ), Toronto Alexithymia Scale (TAS), Visual Analogue Scale (VAS), and Brief Psychological Resilience Scale (BRS). The multivariate analysis utilizing the independent variables—SCS, adaptive and maladaptive CERQ, TAS subscales, BRS, and VAS scores—accounted for 39% of the variance seen in BDI (F (8142) = 11.539, *p* < 0.001). Notably, SCS, adaptive CERQ, and BRS had a negative predictive impact on BDI. Our findings substantiate a statistically significant partial mediatory role of resilience and cognitive emotion regulation in the association between self-compassion and depression. This research accentuates the central role self-compassion, emotional resilience, and cognitive regulation play in the emotional well-being of individuals diagnosed with cancer. Targeted therapeutic interventions focusing on these dimensions may enhance the psychological health of patients, ultimately improving overall treatment outcomes in the oncological setting.

## 1. Introduction

Cancer, a multifaceted illness, emerges and progresses under the influence of not only genetic and environmental factors but also intricate psychological and emotional elements [1]. A major consequence of these intersecting influences is an impact on the immune system, making the patient’s emotional and psychological well-being crucial to the disease’s prognosis. As patients undergo treatment for cancer, a myriad of physical, cognitive, and social alterations become evident [2,3]. These changes range from decreased social functioning and the onset of physical and cognitive disorders to heightened somatic complaints [4,5]. Furthermore, concerns about treatment efficacy and the looming threat of cancer recurrence exacerbate the emotional distress experienced by these patients [1,4,5,6].

While there is a significant volume of research dedicated to psychiatric disorders and the quality of life in cancer patients, studies concerning the emotional processes related to their mental states and potential interventions to bolster emotional strength remain relatively scarce [7]. Recent studies have begun to spotlight self-compassion and stress as pivotal factors that substantially influence the disease’s trajectory [8]. Self-compassion, as conceptualized, represents an emotional regulation mechanism, whereby individuals acknowledge and accept their vulnerabilities, exhibiting kindness, tolerance, and understanding towards themselves [9]. Recognizing suffering as an inherent part of the human experience, individuals must extend care and compassion to themselves amidst their imperfections.

In the realm of psycho-oncology, understanding the multifaceted psychological dimensions of cancer patients has gained paramount importance. While resilience, defined as the ability to “bounce back” and adapt amidst adversity, emerges as a pivotal construct [10], cognitive emotional regulation strategies—how individuals consciously manage and respond to emotionally laden situations—play an equally indispensable role. These strategies can range from re-appraisal and acceptance to suppression, guiding patients in processing and grappling with the intricate nuances of their diagnosis and treatment journey [11].

Alongside these constructs, alexithymia warrants meticulous attention. The term, rooted in the Greek words “a” (none), “lexis” (word), and “thymos” (emotion), epitomizes the difficulty some individuals face in finding words to articulate their emotions [12]. Notably not enlisted in diagnostic manuals such as ICD-10 or DSM-4, alexithymia nevertheless signifies a pervasive psychosomatic phenomenon characterized by impediments in identifying, distinguishing, and conveying emotions. Individuals with alexithymia may be unaware of their emotional states, tending to express their feelings through somatic complaints [13]. Alexithymia has been linked to a variety of psychopathological symptoms including depression, anxiety, somatization, hostility, and paranoia, and can foster a heightened susceptibility to mental disorders, diminish the efficacy of psychotherapy, and negatively influence the development and severity of psychopathological symptoms. Alarmingly, it is reported in approximately 10% of the general populace [14]. A study focusing on cancer patients discovered that over half of the participants exhibited alexithymia, a condition strongly correlated with a more severe perception of the negative outcomes and feelings associated with their illness [15].

Collectively, these elements—resilience, cognitive emotional regulation, and alexithymia—paint a comprehensive portrait of the adaptive and maladaptive psychological tools that could potentially influence the emotional and mental well-being of cancer patients [10,11,13,15].

Understanding the intricate interplay between self-compassion, cognitive emotion regulation, alexithymia, emotional resilience, depression, and anxiety is crucial for safeguarding the psychosocial well-being of cancer patients. In this study, our primary objective was to assess these psychological constructs in cancer patients. Furthermore, we sought to discern the relationships and potential interdependencies among self-compassion, cognitive emotion regulation, alexithymia, emotional resilience, depression, and anxiety. A key focus of our investigation was to determine if emotional resilience and cognitive emotion regulation function as mediators between self-compassion and both depression and anxiety symptoms.

## 2. Materials and Methods

### 2.1. Design, Patients, and Recruitment

This cross-sectional study encompassed a total of 151 patients with a cancer diagnosis. Patients diagnosed with cancer were approached for participation at the Chemotherapy Unit of Gayrettepe Florence Nightingale Hospital during the months of June through August 2023, where they were continuing their chemotherapy regimen. It is noteworthy that while they were at an advanced stage of cancer, they were not in a palliative stage where the focus would be on relieving symptoms and improving quality of life without active anti-cancer treatments. Instead, they were still engaged in active treatment aimed at managing their cancer. To ensure the analysis was not clouded by stage-dependent variations of the disease, only stage 4 cancer patients were included. Eligibility for participation in the study required the individual to be aged 18 years and above, and ready and capable of providing written informed consent. Any patient with a history of psychiatric disorders, dementia, or any other organic neurological disorders was not considered for participation.

### 2.2. Assessment Tools

All eligible and consenting participants underwent an assessment that utilized a sociodemographic data form. Furthermore, several scales and inventories were administered, including Beck Depression Inventory (BDI), Beck Anxiety Inventory (BAI), the Self-Compassion Scale (SCS), Cognitive Emotion Regulation Questionnaire (CERQ), Brief Resilience Scale (BRS), Toronto Alexithymia Scale (TAS-20), and Visual Analogue Scale (VAS).

The Beck Depression Scale (BDI) is a self-assessment tool aiming to gauge the risk and severity of depression in respondents. This 21-item Likert scale delineates depression severity into four distinct categories, ranging from minimal to severe, based on scores between 0 and 63. Validity and reliability studies of BDI for adaptation to the Turkish language have been conducted [16].

Complementing the BDI, the Beck Anxiety Scale (BAI) aids in determining the gravity of anxiety symptoms. Like the BDI, it is structured as a 21-item Likert scale, with the score span stretching from 0 to 63. Studies confirming the validity and reliability of BAI for its Turkish adaptation have been undertaken [17].

Developed by Neff, the Self-Compassion Scale (SCS) measures the intricate facets of self-compassion through six defining sub-dimensions. These sub-dimensions encompass the following: self-kindness vs. self-judgment (this subscale evaluates the extent to which individuals treat themselves with care and understanding versus harsh judgment), common humanity vs. isolation (this gauges the recognition of one’s experiences as part of the larger human experience as opposed to feeling isolated and separated from others), and mindfulness vs. over identification (this dimension assesses the balanced awareness of painful feelings and thoughts, preventing individuals from being consumed by them). The SCS comprises a 26-item questionnaire, with responses anchored on a five-point Likert scale, allowing participants to indicate the extent to which they identify with each statement, providing a comprehensive view of their self-compassion levels. Validity and reliability assessments have been carried out for the Turkish adaptation of SCS [18].

The Cognitive Emotion Regulation Questionnaire (CERQ) is a tool designed to gauge the cognitive strategies individuals utilize to manage emotions. Comprising 36 items dispersed across nine subscales, each item of which pertains to an individual’s thought process subsequent to a distressing occurrence: self-blame (assigning the responsibility of the experience onto oneself), other-blame (attributing the responsibility of the experience to external circumstances or another person), rumination (contemplating the emotions and thoughts associated with the negative incident), catastrophizing (intensely magnifying the severity of the experience), putting into perspective (minimizing the adversity of the event and discerning its significance when juxtaposed with another occurrence), positive refocusing (shifting thoughts towards pleasant subjects rather than ruminating on the event at hand), positive reappraisal (developing a hopeful interpretation of the incident in light of personal growth), acceptance (acknowledging and resigning to the experience), and refocus on planning (strategizing steps and methods to address the adverse occurrence).The usage frequency of each strategy is determined through a 5-point Likert scale, which ranges from 1 (almost never) to 5 (almost always). In this metric, a higher tally in the sub-scale points towards a prevalent usage of that specific strategy. The Turkish version of CERQ maintains established reliability and validity [19]. For analytical clarity, we categorized these strategies into adaptive and maladaptive frameworks. This division was premised on the psychological assertion that cognitive responses can either mitigate or exacerbate emotional distress post-trauma. The adaptive CERQ metric encompasses accumulated scores derived from several sub-scales: acceptance, positive refocusing, planning refocus, positive reappraisal, and perspective analysis. In contrast, the maladaptive CERQ is constituted by the sub-scales: self-blame, rumination, catastrophizing, and blaming others.

The Toronto Alexithymia Scale (TAS-20) is another crucial measure used in this study, specifically tailored to diagnose alexithymia—a condition characterized by difficulties in recognizing and verbalizing one’s emotions. It is a 20-item Likert scale. Within the TAS, there are distinct subscales that focus on specific dimensions of alexithymia: Difficulty Describing Feelings (DDF), Difficulty Identifying Feelings (DIF), and Externally Oriented Thinking (EOT). Each subscale provides further granularity to the assessment, allowing for a comprehensive understanding of the nuances of the alexithymic profile of an individual. Validity and reliability studies of TAS-20 for adaptation to the Turkish language have been conducted [20].

For pain assessments, we resort to the Visual Analogue Scale (VAS). This tool uses a 0–10 cm line, allowing patients to indicate their pain spectrum, from “no pain” to “unbearable pain” [21].

Lastly, the Brief Resilience Scale (BRS) graces our assessment suite. This tool, consisting of six items, is adept at measuring an individual’s aptitude to recuperate and readapt after confronting taxing life events. Assessments for validity and reliability have been conducted for the Turkish adaptation [22].

### 2.3. Statistical Methods

Data analysis was executed utilizing the SPSS 22.0 software suite (IBM Inc., Chicago, IL, USA), which is specifically tailored for social sciences research. We assessed the normality of the distribution of the sample by leveraging skewness and kurtosis values, complemented by the Kolmogorov–Smirnov test. To scrutinize the scale score differences between various subgroups of cancer patients according to the types of their cancer, we utilized the Kruskal–Wallis test. Furthermore, to gauge the interrelations between variables, Pearson’s correlation tests were employed, the application of which was grounded on the data distribution. In our research, we leveraged both univariate and multivariate logistic regression analyses to pinpoint the independent precursors of depression and anxiety in cancer patients. To scrutinize mediation impacts, we employed the PROCESS macro for SPSS, accessible through https://processmacro.org/index.html (accessed on 15 August 2023) [23]. Utilizing a process modeling approach, the endeavor was to delineate the direct relations between self-compassion and both depression and anxiety, while also dissecting the indirect pathways orchestrated through the mediators of emotional resilience and cognitive emotion regulation approaches. The bootstrap method, a non-parametric resampling technique that forgoes the necessity for assuming normality in the sampling distribution, was instituted with a basis of 5000 resamples. To discern the mediation consequences, we engaged bias-corrected 95% confidence intervals (CI). A statistical significance threshold was established at *p* < 0.05. We ensured data completeness by only including patients with fully completed questionnaires in the study; thus, there were no missing data among the eligible patients.

## 3. Results

A total of 151 cancer patients were evaluated in the study. The average age of the patients was 54.94 ± 12.65 years. Descriptive characteristics of the study population are presented in Table 1.

The 151 stage 4 cancer patients exhibited various psychological attributes and states as assessed through an array of standardized scales. On the Beck Depression Inventory (BDI) and Beck Anxiety Inventory (BAI), the participants scored 15.38 ± 4.17 and 11.83 ± 3.00, respectively. Detailed examination through the Self-Compassion Scale (SCS) revealed the following scores across its subscales: self-kindness (2.08 ± 0.59), self-judgment (3.20 ± 0.92), common humanity (2.09 ± 0.41), isolation (2.73 ± 0.76), mindfulness (1.96 ± 0.62), and over-identification (3.54 ± 1.07), culminating in a total SCS score of 14.67 (±2.24) (Table 2).

Exploring the cognitive emotion regulation strategies using the Cognitive Emotion Regulation Questionnaire (CERQ) offered the following scores: self-blame (12.59 ± 4.29), acceptance (8.57 ± 2.32), rumination (14.63 ± 4.19), positive refocusing (7.77 ± 1.83), refocus on planning (9.29 ± 2.59), positive reappraisal (9.36 ± 3.21), putting into perspective (8.86 ± 3.12), catastrophizing (10.72 ± 3.43), and other blame (9.79 ± 2.95). This translated to an adaptive CERQ score of 43.86 ± 6.67 and a maladaptive CERQ score of 47.73 ± 8.69.

Further, the Toronto Alexithymia Scale (TAS) noted scores of 17.29 ± 5.32 for difficulty in describing feelings (DDF), 17.01 ± 5.79 for difficulty in identifying feelings (DIF), and 19.92 ± 3.23 for externally oriented thinking (EOT). Lastly, the participants exhibited a resilience score of 2.11 ± 0.39 as measured through the Brief Resilience Scale (BRS), and perceived pain scored at 6.37 ± 2.30 on the Visual Analogue Scale (VAS).

The Pearson correlation analysis showed that BDI is notably negatively correlated with SCS (r = −0.430, *p* < 0.01) and adaptive CERQ (r = −0.399, *p* < 0.01), but positively correlated with maladaptive CERQ (r = 0.399, *p* < 0.01). Additionally, SCS demonstrated strong negative associations with the sub-scale “overidentification” (r = −0.666, *p* < 0.01) and “self-judgement” (r = −0.571, *p* < 0.01). Other scales and subscales present various levels of significance, with detailed coefficients and significance levels provided in Table 3.

To analyze the factors influencing depression, multiple regression analysis was conducted with BDI as the dependent variable, while SCS, adaptive and maladaptive CERQ, DDT DIF, EOT, BRS, and VAS scores served as independent variables. This model, leveraging SCS, both adaptive and maladaptive CERQ, BRS, and VAS as independent variables, accounts for 39% of the variability in BDI scores (F (8142) = 11.539, *p* < 0.001). Within this array of variables, both maladaptive CERQ and VAS exhibited a positive predictive impact on BDI, while SCS, adaptive CERQ, and BRS had a negative predictive influence (Table 4).

To facilitate the understanding of the tables presented, it is pertinent to elucidate the interpretation of the unstandardized B coefficients depicted. These coefficients signify the magnitude of change to be expected in the dependent variable, which in this context is the BDI scores, correlating with a one-unit alteration in the independent variables while maintaining the other variables at a constant value. Taking an exemplar from the univariate regression analysis, the B value attributed to the SCS stands at −0.798. This indicates that with every unitary increase in the SCS score, there is an anticipated decrease of 0.798 units in the BDI score, under the assumption that all other variables in the equation are held constant. This understanding is vital in decoding the intricate relationships between the different psychological metrics and their combined influence on the BDI scores.

To determine the predictors of anxiety, a multiple regression analysis was executed with BAI designated as the dependent variable and SCS and BRS as the independent variables. This configuration, utilizing SCS and BRS as independent elements, justifies 10% of the variation in BAI scores, according to the equation F (2148) = 8.175, *p* < 0.001. The analysis revealed that both SCS and BRS bear a negative predictive relationship with BAI, as detailed in Table 5.

**Figure 1:**
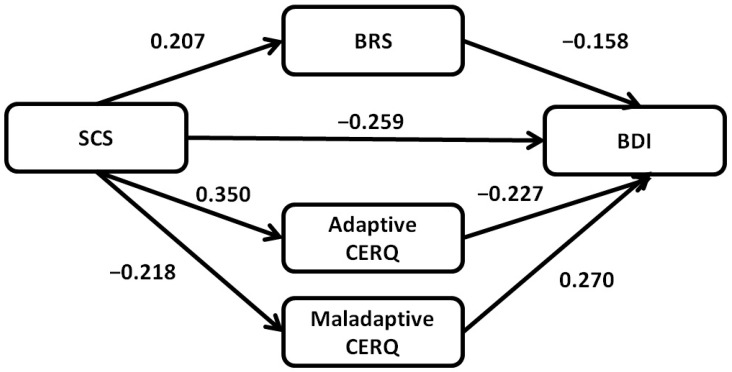
Structural model with hypothesized links between self-compassion, depression, emotional resilience and cognitive emotion regulation with BRS (Brief Resilience Scale), and adaptive and maladaptive CERQ (Cognitive Emotion Regulation Questionnaire) scores as mediators. Values are for standardized coefficients.

To scrutinize the intermediary role of emotional resilience in connecting self-compassion and depression, a mediational examination was undertaken. The mediation model encompassed calculations of both direct and indirect impacts. As depicted in Figure 1, a notable direct influence of self-compassion on depression was discerned (b = −0.482, CI = −0.750 to −0.214, *p* = 0.001), indicating that enhanced self-compassion correlates with diminished depression scores.

Moreover, substantial indirect associations were established both between self-compassion and depression mediated by resilience and between self-compassion and depression mediated by cognitive emotion regulation. These relations emphasize the critical roles that emotional resilience and cognitive emotion regulation strategies undertake in bridging self-compassion and depression, a connection corroborated as statistically substantial in Table 6.

**Figure 2:**
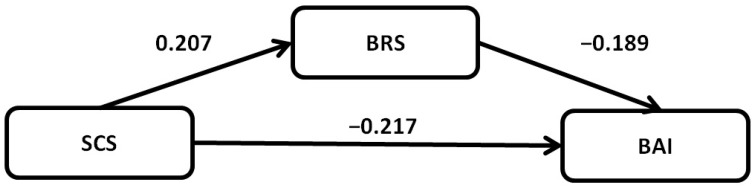
Structural model with hypothesized links between self-compassion, anxiety, and emotional resilience with BRS (Brief Resilience Scale) score as mediator. Values are for standardized coefficients.

Further mediational analysis was carried out to evaluate the intermediary function of emotional resilience in aligning self-compassion and anxiety, with the findings articulated in Table 7. The analysis unearthed significant pathways both direct and indirect, with emotional resilience showcasing a considerable mediating role in this nexus. The partial indirect mediational influence of emotional resilience in the tie between self-compassion and anxiety was established to be statistically noteworthy (Figure 2).

## 4. Discussion

Our study represents a pivotal exploration into the mediating effects of resilience and cognitive emotion regulation strategies on the influence of self-compassion (SC) as a determinant of reduced depression levels in patients diagnosed with a malignancy. Our findings robustly emphasize the significant contribution of SC in mitigating depression severity, reinforcing its validity as a primary predictor in our analysis. Consistent with our hypotheses, self-compassion, resilience, and cognitive emotion regulation strategies emerged as protective bulwarks against depression. Specifically, individuals with cancer diagnoses who exhibited diminished self-compassion, resilience, and adaptive cognitive emotion regulation strategies presented with heightened depression scores. Of note, resilience and cognitive emotion regulation strategies not only demonstrated their own merits as protective factors but also acted as vital mediators in the relationship between SC and depression, as measured by the BDI. Beyond the role of SC, the mediating influences of resilience and cognitive emotion regulation strategies suggest their value as key targets in therapeutic interventions aimed at reducing depression. Furthermore, our findings bolster the case for the integration of compassion-focused interventions in mental health care, especially for those facing profound stressors, such as patients grappling with cancer. Given the marked impact of self-compassion on depression, there’s a compelling argument for embedding compassion-centric modules within therapeutic frameworks to augment their efficacy.

In our examination of cancer types, consistent with the broader literature, breast and lung cancers emerged as the most prevalent [24,25]. The study delved into the psychological turmoil often encountered by individuals grappling with a cancer diagnosis, a scenario that potentially escalates the symptoms of anxiety and depression. This pattern aligns with prior research that showcases the notable emotional turmoil in this group, a consequence of the intricate dynamics associated with the treatment trajectory, persistent fears of relapse, and the existential dilemmas spurred by the affliction [26,27].

The insights gained from the Self-Compassion Scale (SCS) were illuminating. Individuals diagnosed with cancer exhibited diminished scores on SCS subscales, including self-kindness, common humanity, and mindfulness. In contrast, they recorded elevated scores in negative domains like self-judgment, isolation, and over-identification. This deviation accentuates the deleterious psychological sequelae of the illness, where patients might harbor feelings of isolation, augmented self-criticism, and a reduced capacity to remain grounded in the present [28]. Prior studies have emphasized the therapeutic potential of self-compassion in alleviating distress, underscoring its importance in cancer care [29].

Cognitive Emotion Regulation Questionnaire (CERQ), another focal point of the study, showed that cancer patients were more prone to self-blame, rumination, and catastrophizing, while demonstrating lower scores in adaptive strategies like positive refocusing and positive reappraisal. The inclination to catastrophize, coupled with tendencies for self-blame, can augment feelings of despair and powerlessness, further eroding their psychological well-being [11,30,31,32].

Alexithymic traits were investigated among the cancer patients in the study, as evidenced by the scores from the Difficulty in Identifying Feelings (DIF) and Difficulty in Describing Feelings (DDF) components of the Toronto Alexithymia Scale (TAS). These scores indicate potential challenges in recognizing and expressing emotions, which could obstruct effective emotional processing and hinder adaptive coping mechanisms. This is in line with the observations made in the study by Lumley et al., which identified similar challenges in emotional processing [33]. Further research by Yeung et al. underscored the role of alexithymic characteristics such as these in influencing functional well-being, particularly noting the barriers created in emotional processing due to difficulties in identifying and describing feelings [13]. In light of the observed alexithymic traits, implementing emotion-focused therapies might also prove fruitful. By assisting patients in identifying, labeling, and articulating their emotions, the emotional processing can be facilitated, potentially reducing psychological distress. One such therapeutic approach is the Emotion Regulation Group Therapy, which has shown promise in helping individuals with emotion regulation difficulties [34].

In the context of resilience, it transcends mere endurance; it is a repository of protective attributes and personal strengths that facilitate successful adaptation, encompassing elements such as optimism, strong coping mechanisms, and robust social support systems. It is noteworthy that resilience is a dynamic attribute, potentially fluctuating due to a variety of factors including evolving life circumstances and situational challenges. Remarkably, many cancer patients successfully traverse the stressful path of their illness, managing to maintain a semblance of normality in their daily routines and in some instances, experiencing personal growth. It is noteworthy that the journey through cancer can markedly erode this resilience, reflected in the BRS scores of the patients in our study. This erosion of resilience amidst the hardships of the disease accentuates the intricate interplay of physical and psychological challenges they undergo. Not just a testament to the adversities they face, it puts a spotlight on a critical area for support and intervention to enhance their coping mechanisms and improve their quality of life during their cancer journey [10].

A salient aspect of our investigation revolved around the subjective experiences of pain among cancer patients. Pain, both acute and chronic, is frequently intertwined with cancer diagnoses and treatment regimens [35]. Our findings indicated a significant correlation between the intensity of pain and emotional distress markers. Notably, participants reporting higher pain levels often exhibited lower self-compassion scores and relied more heavily on maladaptive cognitive emotional regulation strategies. This suggests that the physical distress of pain may exacerbate emotional turmoil, further underscoring the need for comprehensive pain management in tandem with psychological interventions. It is also crucial to consider the potential bidirectional nature of this relationship, as while pain might heighten emotional distress, maladaptive emotional coping might, in turn, intensify the perception of pain. This intricate relationship calls for a holistic approach to patient care, where pain management and psychological well-being are treated as interconnected domains.

While the study delineates the psychological profile of cancer patients, it also ventures into an unexplored avenue—the potential differences in psychological distress based on the type of malignancy. We observed a distinct variation in BDI scores across various cancer types, with pancreatic cancer patients presenting the most elevated scores, which indicate a higher level of depressive symptoms in this subgroup. This finding prompts a deeper investigation into the underpinnings of these high scores, possibly opening doors to a nuanced understanding of the psychological landscape of cancer patients. It can encourage future research to move towards a more individualized approach to psychological assessment and intervention, allowing for strategies that address not only the general distress experienced by cancer patients but also the specific distress patterns associated with different types of cancer. Recognizing the unique challenges faced by pancreatic cancer patients, in particular, might pave the way for targeted interventions to improve their psychological well-being [36].

Mediational analyses provided crucial insights into the complex relationships among self-compassion, resilience, cognitive emotion regulation, depression, and anxiety. The research affirms the mediating of emotional resilience in navigating the connections between self-compassion and the manifestations of both depression and anxiety. This buttresses the assertion that resilience and self-compassion are intertwined constructs, where fostering self-compassion can bolster resilience, thus ameliorating the mental health outcomes for cancer patients [37].

The pronounced negative coefficient (b = −0.482) in the direct link between self-compassion and depression stands as a significant finding. It indicates a tendency for individuals harboring higher self-compassion to report diminished depression scores, a trait possibly fostered by a gentler self-view and decreased self-reproach, aligning with earlier research narratives. A possible explanation for this could be the core components of self-compassion, which include self-kindness, a sense of shared humanity, and mindfulness [9]. These components enable individuals to treat themselves with kindness, understand their struggles as part of the broader human experience, and observe their feelings non-judgmentally. Collectively, these elements could counteract the feelings of isolation, rumination, and self-criticism that often accompany depression.

Similarly, cognitive emotion regulation strategies, which encompass how individuals think about their emotions and how they handle them, can be influenced by self-compassion. A self-compassionate mindset might deter maladaptive cognitive strategies, such as rumination or catastrophizing, which are often implicated in depressive symptomology. Instead, individuals might be encouraged to adopt more adaptive strategies, like positive reappraisal or acceptance. Fostering a self-compassionate approach could potentially curb the reliance on maladaptive cognitive processes like rumination or catastrophizing, frequently associated with depressive symptoms. Conversely, it might promote the adoption of adaptive strategies such as positive reappraisal or acceptance, steering individuals toward a healthier mental state.

The mediational model for anxiety mirrored that of depression, further solidifying the protective role of self-compassion. The indirect effects point to emotional resilience as a potential buffer against anxiety, positing that self-compassionate individuals may develop stronger emotional coping mechanisms that reduce anxiety susceptibility.

While scant research has probed into the interplay of self-compassion, resilience, and cognitive emotion regulation within the cancer patient demographic, these concepts resonate deeply in broader populations. Neff’s depiction of self-compassion positions it as a robust shield against depression and anxiety, corroborated by studies like that of Van Dam et al. [9,38]. The literature distinctly underscores the tandem effect of mindfulness and self-compassion in assuaging emotional distress, propelling resilience to the forefront as a potential mediating agent [39]. Resilience, framed as the innate human capacity to rebound from setbacks, melds seamlessly with mindfulness, and stands as a cornerstone in well-being outcomes [40]. Preliminary links suggest a deep-rooted symbiosis between resilience and self-compassion, although further exploration remains due [41].

Reflecting upon the extant literature and the revelations from our investigation, it is evident that self-compassion, whether directly or mediated via resilience, cast a profound impact on emotional states like anxiety and depression. Remarkably, consistent with earlier anticipations, our analysis amplifies the unique potency of self-compassion, suggesting it might even overshadow mindfulness in orchestrating emotional equilibrium. Such insights accentuate the imperativeness of weaving mindfulness, self-compassion, and resilience into therapeutic paradigms, fostering a comprehensive upliftment in patient wellness trajectories.

### 4.1. Clinical Implications

The rising global incidence of cancer, coupled with increased mortality rates for certain malignancies, underscores the persistent challenges faced by the community in the comprehensive management of cancer. Against this backdrop, the findings from our study take on heightened importance, offering pivotal clinical insights, particularly in the field of oncological therapy [42]. The intricate relationship between self-compassion, emotional resilience, and cognitive emotional regulation strategies offers a roadmap for clinicians to structure targeted interventions. In particular, the elevated prevalence of maladaptive emotion regulation strategies among cancer patients suggests an immediate need for interventions that prioritize cognitive restructuring and emotion-focused coping. Clinicians might consider incorporating modules on enhancing self-awareness and emotional recognition, given the challenges associated with alexithymia. Furthermore, the evident benefits of self-compassion as a buffer against emotional distress underline its potential as a therapeutic tool. Healthcare practitioners may also need to take into account the heterogeneity of cancer experiences; individualized interventions that cater to the specific emotional needs and profiles of patients may prove more efficacious than one-size-fits-all approaches. Given the significant mediational roles of emotional resilience and cognitive emotion regulation, interventions that bolster these components could be especially beneficial for individuals prone to depression and anxiety. For instance, resilience training programs or cognitive behavioral therapy, which focus on building resilience and refining cognitive emotion regulation strategies, respectively, could be particularly effective. These interventions, combined with practices that nurture self-compassion, like mindfulness-based stress reduction or compassion-focused therapy, could offer a multifaceted approach to mental well-being. By offering techniques to cope with adversity and maintain emotional equilibrium, these programs can serve as a crucial pillar of support for cancer patients.

While the current study provides essential insights into the psychological landscape of cancer patients, several avenues remain to be explored. Longitudinal studies can shed light on the evolving psychological dynamics over different stages of the cancer journey—from diagnosis to treatment and potential remission or recurrence. Such investigations can chart the ebb and flow of psychological parameters, enabling a more comprehensive understanding. Additionally, exploring potential moderating variables, like social support, types of cancer, and stages of cancer, can offer a more nuanced perspective. The potential protective role of factors like familial support, community involvement, or even spiritual beliefs, in the face of a cancer diagnosis, remains a fertile ground for exploration.

### 4.2. Limitations

The study, though insightful, bears several potential limitations that warrant consideration. First, its cross-sectional design precludes the establishment of definitive causality, making longitudinal studies a more desirable avenue for verifying directional relationships among self-compassion, emotional resilience, cognitive emotion regulation, depression, and anxiety. The reliance on self-report measures poses the risk of introducing biases, as participants may not consistently offer accurate or forthright responses, potentially influenced by social desirability or an imprecise self-awareness. Moreover, the findings’ applicability may be circumscribed by the sample characteristics, especially if lacking diversity in age, ethnicity, socioeconomic status, or other pertinent demographics. Additionally, the operational definitions and measurements for self-compassion, emotional resilience, and cognitive emotion regulation might not wholly encapsulate the breadth and nuances of these intricate constructs, leaving room for potential oversights or cultural biases. Unexamined confounding variables, such as past traumas, present-day stressors, or support structures, might unduly affect the delineated relationships. Furthermore, while emotional resilience and cognitive emotion regulation are spotlighted as mediators, other latent factors could also play a pivotal role but remain unexplored. External elements like societal or cultural dynamics, which might interplay with the central variables, may not be adequately considered. Without tangible experimental data, extrapolating actionable therapeutic interventions from merely observed correlations remains a challenge.

## 5. Conclusions

The intricate relationships between self-compassion, emotional resilience, cognitive emotion regulation, depression, and anxiety offer profound insights into the psychological mechanisms at play. Recognizing the mediating roles of resilience and emotion regulation not only enriches our understanding but also points towards targeted therapeutic strategies that can significantly enhance mental health outcomes.

## Figures and Tables

**Table 1:** Sociodemographic and clinical features.

	Cancer Patients (*n* = 151)
Age	54.94 ± 12.65
Sex	
Female	92 (60.9)
Male	59 (39.1)
Marital status	
Single/Widowed/divorced	38 (25.2)
Married	113 (74.8)
Education	
Primary school	23 (15.2)
Secondary school	19 (12.6)
High school	51 (33.8)
College	46 (30.5)
PhD	12 (7.9)
Types of cancer	
Breast cancer	41 (27.2)
Lung cancer	35 (23.2)
Colorectal cancer	18 (11.9)
Pancreatic cancer	15 (9.9)
Gastric cancer	13 (8.6)
Nasopharyngeal and laryngeal cancer	8 (5.3)
Other *	21(13.9)

Numbers indicate mean ± standard deviation or n (%). * Ovarian, prostate, endometrial cancer; cholangiocarcinoma.

**Table 2:** Scores from Beck Depression Inventory (BDI), Beck Anxiety Inventory (BAI), Self-Compassion Scale (SCS), Cognitive Emotion Regulation Questionnaire (CERQ), Toronto Alexithymia Scale-20 subscales DIF (difficulty in identifying feelings), DDF (difficulty in describing feelings), EOT (externally oriented thinking), Brief Resilience Scale (BRS), and Visual Analog Scale (VAS).

	Cancer Patients (*n* = 151)
BDI	15.38 ± 4.17
BAI	11.83 ± 3.00
SCS self-kindness	2.08 ± 0.59
SCS self-judgement	3.20 ± 0.92
SCS common humanity	2.09 ± 0.41
SCS isolation	2.73 ± 0.76
SCS mindfulness	1.96 ± 0.62
SCS over identification	3.54 ± 1.07
SCS	14.67 ± 2.24
CERQ self-blame	12.59 ± 4.29
CERQ acceptance	8.57 ± 2.32
CERQ rumination	14.63 ± 4.19
CERQ positive refocusing	7.77 ± 1.83
CERQ refocus on planning	9.29 ± 2.59
CERQ positive reappraisal	9.36 ± 3.21
CERQ putting into perspective	8.86 ± 3.12
CERQ catastrophizing	10.72 ± 3.43
CERQ other blame	9.79 ± 2.95
adaptive CERQ	43.86 ± 6.67
maladaptive CERQ	47.73 ± 8.69
DDF	17.29 ± 5.32
DIF	17.01 ± 5.79
EOT	19.92 ± 3.23
BRS	2.11 ± 0.39
VAS	6.37 ± 2.30

Numbers indicate mean ± standard deviation.

**Table 3:** Pearson correlation analysis among assessed scale scores and subscores, including Beck Depression Inventory (BDI), Beck Anxiety Inventory (BAI), Self-Compassion Scale (SCS), Cognitive Emotion Regulation Questionnaire (CERQ), Toronto Alexithymia Scale-20 subscales DIF (difficulty in identifying feelings), DDF (difficulty in describing feelings), EOT (externally oriented thinking), Brief Resilience Scale (BRS), and Visual Analog Scale (VAS).

	BDI	BAI	SCS	Adaptive CERQ	Maladaptive CERQ	BRS	VAS
BDI	1	0.197 *	−0.430 **	−0.399 **	0.399 **	−0.267 **	0.209 **
BAI	0.197 *	1	−0.256 **	−0.149	0.087	−0.233 **	0.151
SCS	−0.430 **	−0.256 **	1	0.350 **	−0.218 **	0.205 *	−0.099
SCS self-kindness	−0.253 **	−0.032	0.385 **	0.192 *	−0.211 **	0.059	0.020
SCS self-judgement	0.224 **	0.134	−0.571 **	−0.116	−0.039	−0.120	−0.079
SCS common humanity	−0.204 *	−0.227 **	0.367 **	0.127	−0.131	0.215 **	−0.023
SCS isolation	0.164 *	0.167 *	−0.468 **	−0.084	0.186 *	−0.114	0.032
SCS mindfulness	−0.273 **	−0.271 **	0.436 **	0.264 **	−0.182 *	−0.039	−0.206 *
SCS over identification	0.215 **	0.041	−0.666 **	−0.269 **	0.086	−0.152	0.135
CERQ self−blame	0.280 **	0.077	−0.245 **	−0.106	0.669 **	−0.171 *	0.078
CERQ acceptance	−0.230 **	−0.116	0.243 **	0.502 **	−0.194 *	0.001	−0.078
CERQ rumination	0.237 **	−0.006	−0.133	−0.234 **	0.587 **	−0.072	−0.016
CERQ positive refocusing	−0.223 **	−0.149	0.272 **	0.329 **	−0.179 *	0.137	−0.164 *
CERQ refocus on planning	−0.215 **	−0.023	0.170 *	0.493 **	−0.104	0.053	−0.061
CERQ positive reappraisal	−0.191 *	−0.035	0.185 *	0.595 **	−0.052	0.123	0.017
CERQ putting into perspective	−0.175 *	−0.0 adaptive CERQ89	0.077	0.549 **	−0.124	−0.026	−0.069
CERQ catastrophizing	0.194 *	0.103	0.011	−0.088	0.567 **	0.027	0.182 *
CERQ other blame	0.206 *	0.033	−0.110	−0.119	0.479 **	−0.029	−0.096
DDF	0.230 **	−0.005	−0.155	−0.201 *	0.150	−0.176 *	0.014
DIF	0.198 *	−0.045	−0.095	−0.096	0.074	−0.051	0.015
EOT	0.246 **	0.039	−0.159	−0.280 **	0.189 *	−0.036	0.167 *

* *p* < 0.05, ** *p* < 0.01.

**Table 4:** Univariate and multivariate regression analysis of Self-Compassion Scale (SCS), Cognitive Emotion Regulation Strategies (CERQ), Toronto Alexithymia Scale-20 subscales DIF (difficulty in identifying feelings), DDF (difficulty in describing feelings), EOT (externally oriented thinking), Brief Resilience Scale (BRS), Visual Analog Scale (VAS), and Beck Depression Inventory (BDI) scores.

Univariate Regression Analysis									
	Unstandardized				Standardized				
	B	SE	Lower	Upper	β	t	R2	F	*p* Value
SCS	−0.798	0.137	−1.070	−0.527	−0.430	−5.807	0.179	33.723	<0.001
adaptive CERQ	−0.249	0.047	−0.342	−0.156	−0.399	−5.308	0.159	28.170	<0.001
negative CERQ	0.191	0.036	0.120	0.262	0.399	5.317	0.159	28.270	<0.001
DDF	0.180	0.062	0.056	0.303	0.230	2.880	0.053	8.293	0.005
DIF	0.143	0.058	0.029	0.257	0.198	2.470	0.039	6.100	0.015
EOT	0.317	0.102	0.114	0.519	0.246	3.092	0.060	9.562	0.002
BRS	−2.819	0.835	−4.468	−1.170	−0.267	−3.378	0.071	11.408	0.001
VAS	0.378	0.145	0.092	0.664	0.209	2.610	0.044	6.810	0.010
**Multivariate regression analysis**									
	**Unstandardized**				**Standardized**				
	B	SE	Lower	Upper	β	t	R2	F	*p*−value
SCS	−0.440	0.134	−0.704	−0.176	−0.237	−3.292	0.394	11.539	0.001
adaptive CERQ	−0.116	0.046	−0.207	−0.026	−0.186	−2.539			0.012
maladaptive CERQ	0.119	0.033	0.053	0.184	0.248	3.595			<0.001
DDF	0.055	0.054	−0.052	0.161	0.070	1.017			0.311
DIF	0.081	0.048	−0.014	0.177	0.113	1.688			0.094
EOT	0.058	0.091	−0.123	0.239	0.045	0.637			0.525
BRS	−1.609	0.716	−3.024	−0.194	−0.152	−2.247			0.026
VAS	0.251	0.121	0.012	0.489	0.139	2.079			0.039

Beck Anxiety Inventory (BAI), Beck Depression Inventory (BDI), Brief Resilience Scale (BRS), Cognitive Emotion Regulation Questionnaire (CERQ), DDF (difficulty in describing feelings), DIF (difficulty in identifying feelings), EOT (externally oriented thinking), Self-Compassion Scale (SCS), and Visual Analog Scale (VAS).

**Table 5:** Univariate and multivariate regression analysis of Self-Compassion Scale (SCS), Cognitive Emotion Regulation Strategies (CERQ), Toronto Alexithymia Scale-20 subscales DIF (difficulty in identifying feelings), DDF (difficulty in describing feelings), EOT (externally oriented thinking), Brief Resilience Scale (BRS), Visual Analog Scale (VAS), and Beck Anxiety Inventory (BAI) scores.

Univariate Regression Analysis									
	Unstandardized				Standardized				
	B	SE	Lower	Upper	β	t	R2	F	*p* value
SCS	−0.343	0.106	−0.553	−0.133	−0.256	−3.226	0.065	10.409	0.002
pCERQ	−0.067	0.037	−0.139	0.005	−0.149	−1.834	0.022	3.364	0.069
nCERQ	0.030	0.028	−0.026	0.086	0.087	1.071	0.008	1.148	0.286
DDF	−0.003	0.046	−0.095	0.089	−0.005	−0.065	<0.001	0.004	0.949
DIF	−0.023	0.043	−0.107	0.061	−0.045	−0.549	0.002	0.301	0.584
EOT	0.036	0.076	−0.114	0.187	0.039	0.478	0.002	0.228	0.633
BRS	−1.781	0.608	−2.984	−0.579	−0.233	−2.928	0.054	8.574	0.004
VAS	0.197	0.106	−0.012	0.406	0.151	1.867	0.023	3.484	0.064
**Multivariate regression analysis**									
	**Unstandardized**				**Standardized**				
	B	SE	Lower	Upper	β	t	R2	F	*p* value
SCS	−0.291	0.107	−0.503	0.080	0.217	−2.722	0.10	8.175	0.007
BRS	−1.443	0.609	−2.645	0.240	0.189	−2.370			0.019

**Table 6:** Mediation Analysis of Self-Compassion Scale (SCS), Adaptive Strategies Component of Cognitive Emotion Regulation Questionnaire (aCERQ), Maladaptive Strategies Component of Cognitive Emotion Regulation Questionnaire (mCERQ), Brief Resilience Scale (BRS), and Beck’s Depression Inventory (BDI) Scores.

	Effect	Standardized Error	Standardized Coefficient	*t*	Lower	Upper	*p*
**COMPONENTS**							
SCS−>BRS	0.036	0.014	0.207	2.551	0.008	0.064	0.012
SCS−>aCERQ	1.042	0.228	0.350	4.567	0.591	1.494	<0.001
SCS−>mCERQ	−0.846	0.310	−0.218	−2.728	−1.458	−0.233	0.007
BRS−>BDI	−1.668	0.722	−0.158	−2.310	−3.095	−0.241	0.022
aCERQ−>BDI	−0.141	0.045	−0.227	−2.130	−0.231	−0.052	0.002
mCERQ−>BDI	0.129	0.033	0.270	3.874	0.063	0.195	<0.001
**INDIRECT EFFECT**							
H1: SCS−>BRS−>BDI	−0.060	0.036	−0.032		−0.141	−0.003	
H2: SCS−>aCERQ−> BDI	−0.147	0.065	−0.079		−0.294	−0.042	
H3: SCS−>mCERQ−>BDI	−0.109	0.048	−0.059		−0.212	−0.020	
**DIRECT EFFECT**							
SCS−>BDI	−0.482	0.136	−0.259	−3.551	−0.750	−0.214	0.001
**TOTAL EFFECT**							
SCS−>BDI	−0.798	0.138	−0.430	−5.807	−1.070	−0.527	<0.001

**Table 7:** Mediation Analysis of Self-Compassion Scale (SCS), Brief Resilience Scale (BRS), and Beck’s Anxiety Inventory (BAI) Scores.

	Effect	Standardized Error	Standardized Coefficient	*t*	Lower	Upper	*p*
**COMPONENTS**							
SCS−>BRS	0.036	0.014	0.207	2.551	0.008	0.064	0.012
BRS−>BAI	−1.443	0.609	−0.189	−2.370	−2.645	−0.240	0.019
**INDIRECT EFFECT**							
SCS−>BRS−>BAI	−0.052	0.031	−0.039		−0.123	−0.003	
**DIRECT EFFECT**							
SCS−>BAI	−0.291	0.107	−0.217	−2.722	−0.503	−0.080	0.007
**TOTAL EFFECT**							
SCS−>BAI	−0.343	0.106	−0.256	−3.226	−0.553	−0.133	0.002

## Data Availability

Data supporting the findings of this study are available upon reasonable request from the corresponding author.
